# Explore the value of carotid ultrasound radiomics nomogram in predicting ischemic stroke risk in patients with type 2 diabetes mellitus

**DOI:** 10.3389/fendo.2024.1357580

**Published:** 2024-04-19

**Authors:** Yusen Liu, Ying Kong, Yanhong Yan, Pinjing Hui

**Affiliations:** Department of Stroke Center, The First Affiliated Hospital of Soochow University, Suzhou, Jiangsu, China

**Keywords:** type 2 diabetes mellitus, ischemic stroke, carotid atherosclerotic plaque, triglyceride-glucose index, carotid ultrasound, radiomics, machine learning, nomogram

## Abstract

**Background and objective:**

Type 2 Diabetes Mellitus (T2DM) with insulin resistance (IR) is prone to damage the vascular endothelial, leading to the formation of vulnerable carotid plaques and increasing ischemic stroke (IS) risk. The purpose of this study is to develop a nomogram model based on carotid ultrasound radiomics for predicting IS risk in T2DM patients.

**Methods:**

198 T2DM patients were enrolled and separated into study and control groups based on IS history. After manually delineating carotid plaque region of interest (ROI) from images, radiomics features were identified and selected using the least absolute shrinkage and selection operator (LASSO) regression to calculate the radiomics score (RS). A combinatorial logistic machine learning model and nomograms were created using RS and clinical features like the triglyceride-glucose index. The three models were assessed using area under curve (AUC) and decision curve analysis (DCA).

**Results:**

Patients were divided into the training set and the testing set by the ratio of 0.7. 4 radiomics features were selected. RS and clinical variables were all statically significant in the training set and were used to create a combination model and a prediction nomogram. The combination model (radiomics + clinical nomogram) had the largest AUC in both the training set and the testing set (0.898 and 0.857), and DCA analysis showed that it had a higher overall net benefit compared to the other models.

**Conclusions:**

This study created a carotid ultrasound radiomics machine-learning-based IS risk nomogram for T2DM patients with carotid plaques. Its diagnostic performance and clinical prediction capabilities enable accurate, convenient, and customized medical care.

## 1 Introduction

Ischemic stroke (IS) is a condition characterized by a disruption in blood flow to brain tissue due to various circumstances. It is a significant global cause of mortality and frequently results in disabling events (1). Atherosclerosis in large arteries is the primary cause of cerebral infarction, which is the common cause of stroke (2). Type 2 diabetes mellitus (T2DM), characterized by insulin resistance (IR) as the main underlying mechanism, is not only leading to the damage of vascular endothelial and consequently leading to the formation and rupture of plaques in the carotid artery (3–5), but also contributes to the occurrence, recurrence, disability, and mortality of IS (6).

Individuals with T2DM are at an elevated risk of developing carotid plaques. Numerous factors have been linked to the development of atherosclerosis in individuals with T2DM, including IR, inflammation, age, obesity, tobacco use, dyslipidemia, and other stress markers (7, 8). Furthermore, diabetic dyslipidemia in individuals with T2DM is marked by elevated triglycerides (TG) and low-density lipoprotein cholesterol (LDL-C) levels, alongside diminished high-density lipoprotein cholesterol (HDL-C) levels. This condition has been associated with heightened susceptibility to plaque formation and hastened development of atherosclerosis in T2DM patients. Recent studies pointed out that multiple molecular mechanisms are involved in the formation of carotid vulnerable plaques. For example, the nucleotide-binding oligomerization domain-like receptor protein 3 (NLRP3) inflammasome, a vital component of the innate immune system, can be activated by different stimulation, including ATP, Toll-like receptor ligands, mitochondrial dysfunction, the production of reactive oxygen species, and ionic flux (9), eventually orchestrating lipid-driven amplification of vascular inflammation, promoting the disruption of the fibrous cap (10). At the same time, abnormal indoleamine 2,3-dioxygenase 1 (IDO1)-regulated tryptophan metabolism promotes osteogenic reprogramming and calcification in vascular smooth muscle cells (11), whereas surface calcification is a key hallmark of carotid susceptible plaque.

Previously, the measurement of carotid artery stenosis was used to assess the risk of stroke (12). However, recent evidence suggests that it is crucial to consider not only the degree of carotid artery stenosis, but also the connection between vulnerable plaques and the occurrence of IS (13). T2DM together with vulnerable carotid plaque and carotid stenosis, lead to an increased risk of IS. It is crucial to accurately quantify the risk of stroke occurrences in T2DM patients in order to provide tailored diagnosis and therapy.

Carotid ultrasound (CDU) is a widely used tool for evaluating vulnerable plaques in carotid vascular, due to its low cost, short time consumption, non-invasiveness, and non-radiation damage to the human body when compared to magnetic resonance imaging (MRI) or computed tomography angiography (CTA) (14, 15). CDU is suitable for the observation of the morphological changes of the vascular wall and determine the nature of the carotid plaques. It can also observe the hemodynamic changes in the lumen and determine the vascular stenosis or occlusion. Vulnerable carotid plaques can be identified by CDU by assessing the shape, size, ulceration, rupture, and other characteristics of the plaque (14, 16–18). Images of carotid vascular plaques in T2DM patients can be easily obtained to further investigate their impact on IS.

The triglyceride-glucose index (TyG index) is a reliable indication for assessing insulin resistance (IR) (19). The TyG index is calculated only by fasting blood sugar (FBS) and TG, two blood indexes that are easily obtained (20). Consequently, numerous studies suggested that it is a simpler and more effective diagnostic tool for IR compared to prior techniques of detection (21, 22). Prior research had established a correlation between a higher TyG index and increased vulnerability of carotid artery plaque in non-diabetic persons (23). TyG index was also related to IS, IR plays a significant role in the development of IS through a range of potential mechanisms (24). Meanwhile, the TyG index serves as an indicator of IR, offering an indirect means to predict to the occurrence of IS. A previous meta-analysis demonstrated that an increased TyG index was an independent risk factor for IS (25). Therefore, the TyG index can be used to assess the risk of IS in patients with T2DM.

Radiomics is a newly emerging research method that converts medical images into high-throughput data that can be used to extract and analyze image information that cannot be recognized by the human eye, and quantitative relationships between medical images and diseases can be obtained by combining machine learning (ML) methods to build a diagnostic model (26). Radiomics can be performed on CT, MRI, and ultrasound images, and texture-based research has become one of the hot spots of radiomics research. Previous research by our team showed that the textural analysis of carotid plaques can be used to determine plaque vulnerability (27).

Currently, there are only a limited number of radiomics models that assess the risk of IS in individuals with T2DM. Thus, this study utilized radiomics analysis methods of carotid plaques using CDU images, along with clinical indicators such as the TyG index, to develop a nomogram model for predicting the risk of IS in patients with T2DM, which can aid in personalized and stratified diagnosis and treatment of T2DM patients.

## 2 Materials and methods

### 2.1 Patients inclusion

**Figure 1:**
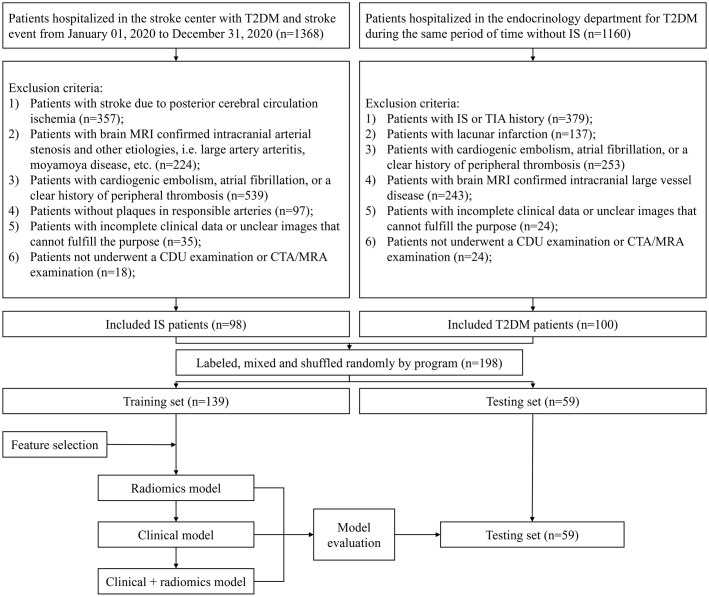
Inclusion exclusion flowchart.

The study group included T2DM patients who had strokes and were hospitalized at the First Affiliated Hospital of Soochow University Stroke Centre between January 1 and December 31, 2020. Inclusion criteria were: (1) Clinically confirmed IS by MRI; (2) CDU inspection confirmed plaque formation or even stenosis in the carotid artery on the responsible side. Exclusion criteria were: (1) Patients with stroke due to posterior cerebral circulation ischemia; (2) Patients with brain MRI confirmed intracranial arterial stenosis and other etiologies, i.e. large artery arteritis, moyamoya disease, etc.; (3) Patients with cardiogenic embolism, atrial fibrillation, or a clear history of peripheral thrombosis; (4) Patients without plaques in responsible arteries; (5) Patients with incomplete clinical data or unclear images that cannot fulfill the purpose. Control group includes T2DM patients admitted to endocrinology for treatment throughout the same time period. The inclusion criteria were: (1) Clinically recognized as T2DM; (2) CDU examination confirmed the existence of the carotid plaques or even stenosis. Exclusion criteria were: (1) Patients with IS or TIA history; (2) Patients with lacunar infarction; (3) Patients with a history of coronary artery disease or a clear history of peripheral thrombosis; (4) Patients with MRI confirmed intracranial large vessel disease; (5) Patients with incomplete clinical data or unclear imaging images that cannot be fulfilled for the purpose of imaging histology extraction. A total of 198 patients were included in the study. The study group had 98 and the control group 100. Following randomization, 139 patients entered the training group and 59 entered the testing group. Clinical data included age, sex, smoking/alcohol history, living place, LDL-C, HDL-C, TG, total cholesterol (TC), FBS, and TyG index calculated from FBS and TG. The detailed flow chart is shown in **Figure 1**. The procedures had been reviewed and approved by the medical ethics committee of the First Affiliated Hospital of Soochow University [Audit number: (2023) No.132].

### 2.2 Clinical data collection and calculation

Among the basic clinical information of patients, smoking was defined as at least one cigarette per day for more than 1 year in the last 10 years (after 2009). Drinking was defined as alcohol intake of at least 90/45g per day for male/female in the past 10 years (after 2009). Living area was defined as city or countryside; and body mass index (BMI) was calculated as weight (kg) divided by the square of height (m^2^). Seated blood pressure was measured 3 times and averaged using a mercury column sphygmomanometer, calculated as systolic blood pressure (SBP) and diastolic blood pressure (DBP), pulse pressure (PP) was defined as the differential of SBP and DBP. Laboratory index were tested from 3 to 5mL of fasting plasma obtained from the patient’s anterior elbow vein after an 8-12 hour overnight fast, which included FBS, TC, TG, LDL-C and HDL-C levels, all tests were performed in the central laboratory of the First Affiliated Hospital of Soochow University using an automated analyzer. TyG index was calculated as ln(TG (mg/dL)×FBG (mg/dL)/2) (20)

The degree of carotid artery stenosis was assessed by CDU, according to North American Symptomatic Carotid Endarterectomy Trial (NASCET). Patients with no stenosis (label=0) were defined as those with normal tube diameter without stenosis, mild stenosis (label=1) was defined as artery stenosis with a stenosis rate between 0% and 49%, moderate stenosis (label=2) was defined as artery stenosis with a stenosis rate between 50% and 69%, and severe stenosis (label=3) was defined as artery stenosis with a stenosis rate between 70% to 99% (12). Vulnerable plaques were characterized as heterogeneous, hypoechoic or moderately hypoechoic plaques on CDU, with an irregular surface shape, incomplete fibrous cap or intra-plaque blood flow signals on color Doppler ultrasound (ulcerative plaques) (17), as well as surface calcification, multiple calcification and ulceration of atherosclerotic plaques (18).

### 2.3 Plaque segmentation and feature extraction

**Figure 2:**
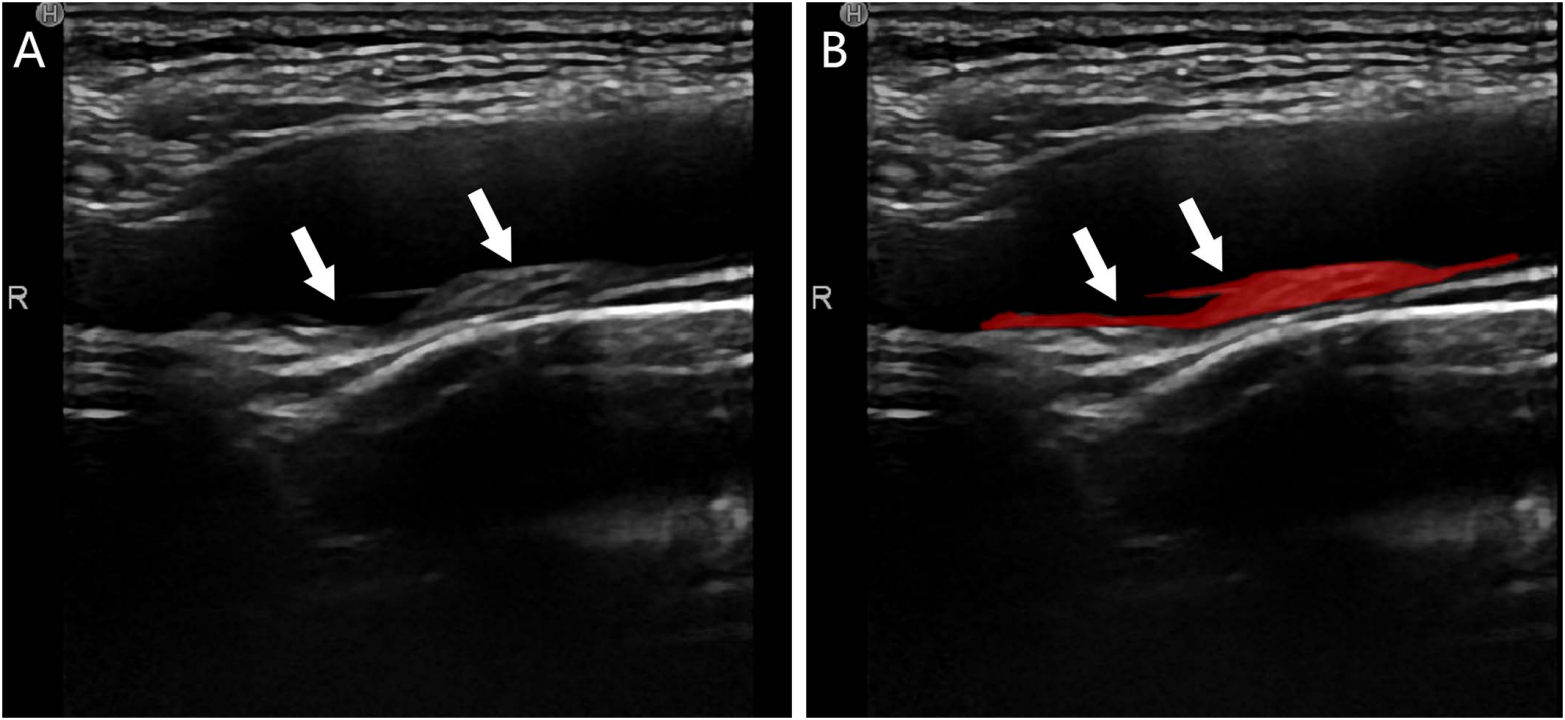
Plaque segmentation schematic of a T2DM patient, male, 55 years old, with no stroke history; **(A)** CDU showed a mixed-echo inhomogeneous plaque extending from the right the bulb of the right carotid artery to the internal carotid artery (white arrows) suggestive of a vulnerable plaque; **(B)** ROI delineation of the plaque by the observer (red area shown with white arrows).

CDU images of the carotid arteries of all patients were acquired in DICOM format from the picture archiving and communication system (PACS) of the institution. Region of interest (ROI) was manually determined using ITK-SNAP 4.0.1 software (28). In order to avoid influence from insufficient picture contrast, images of 198 patients were normalized with MATLAB R2020 to distribute pixel grey values between 0 and 1. 2 senior ultrasonography physicians (observers) blinded to clinical results manually established ROIs based on the longitudinal CDU’s maximal plaque area (**Figure 2**). Gray-scale normalization was performed between m ± 3d (m = ROI grey level mean; d = standard deviation) to mitigate the effects of data acquisition environment, parameters, and other factors on grey scale images (27). This technique improves experimental comparability and reliability, as shown by prior study (29, 30).

7 feature groups (including 98 radiomics features), including shape feature (2D) (5 features), first-order statistics (18 features), gray level dependence matrix (GLDM, 14 features), gray level co-occurrence matrix (GLCM, 24 features), gray level run length matrix (GLRLM, 16features), gray level size zone matrix (GLSZM, 16 features), neighboring gray tone difference matrix (NGTDM,5 features), were extracted by using PyRadiomics package based on Python 3.10 (31), the definitions and the details of these features are shown in https://pyradiomics.readthedocs.io/en/latest/index.html.

Radiomics feature extraction returned 98 features (Shown in **Supplementary Table ICC**). Z-score normalization was used to lessen the impact of significant outliers or variables with substantial magnitude differences on the results. A total of 40 images (20 from each of the two groups) were utilized to evaluate radiomics characteristics’ intra- and inter-observer agreement to ensure data interpretation consistency. Two independent ultrasound specialists (observer 1 and observer 2) identified the ROI on each group of 20 images for inter-observer analysis. Clinical results were unknown to them. For intra-observer reliability, observer 1 performed ROI delineation and radiomics feature extraction on 40 images after 2 weeks. The ROI definition was then completed for all images. The intraclass correlation coefficient (ICC) assessed variable reliability. Features having an ICC value above 0.75 were retained for model analysis due to their high dependability, while those having ICC value lower than 0.75 were excluded.

### 2.4 Radiomics feature selection, ML model selection and Rad-score calculation

T-test analysis was performed to find statistically significant (*p*-value < 0.05) radiomics features in the training set. These features were utilized to construct and evaluate three ML models: Support Vector Classification (SVC), Random-Forest Classifier (RF), and Logistic Regression (LR). The effectiveness was evaluated and judged using receiver operating characteristic curves (ROC) and the detailed procedures are provided in the Supplementary materials (**Supplementary Figure 1**). In the end, LR ML model was then selected for constructing the subsequent model.

The radiomics features that passed the T-test were then included in the Least Absolute Shrinkage and Selection Operator (LASSO) regression (32), which selected features with non-zero coefficients to distinguish the study group from the control group (33, 34). Tune the regularization parameter λ to govern the magnitude of regularization. The optimal λ value was determined using 10-fold cross-validation and the 1-standard error of the minimal criteria (the 1-SE criteria). The features with non-zero coefficients were ultimately incorporated and assigned weights based on their coefficients in LASSO regression. This process could result in the generation of a radiomics score (RS) for each patient.

### 2.5 Clinical feature selection and ML models construction and evaluation

The clinical data were partitioned into training and testing sets based on the corresponding sets of radiomics. T-test or ANOVA analysis was conducted based on the normality of the clinical data in the training set. Only those with a *p*-value less than 0.05 were considered. Multivariate LR analysis was conducted on these variables to identify clinical features with statistical significance.

The LR ML model was used to build three ML models. The clinical features described above were used to build clinical LR ML models. The radiomics training set data were then utilized to develop a radiomics LR ML model employing the radiomics features with a non-zero coefficient in LASSO regression. Finally, the clinical + radiomics model was created, which incorporated both the clinical and radiomics features.

The three models’ ROC curves were calculated to evaluate their performance. The three ML models’ prediction performance in the training and testing sets was assessed by the area under curve (AUC) size. The training set calculated the net benefit rate using decision curve analysis (DCA) at various threshold probabilities. SHapley Additive exPlanations (SHAP) visualization of selected clinical features and RS was applied to visually measure the predictive power of each feature by its horizontal range.

### 2.6 Radiomics nomogram construction

A radiomics nomogram score (Nomo-score) was calculated for each patient using the constructed clinical + radiomics LR ML model. A predictive nomogram model was then constructed. Additionally, calibration curves were created and evaluated separately for the training set and the test set to evaluate the performance of the Nomogram, the brier score was also calculated and evaluated.

### 2.7 Data analysis

SPSS v.26.0 (SPSS Inc., Chicago, IL, USA), R statistical software (v.4.3.0; https://www.r-project.org) and python 3.10.0 were used for statistical analysis. For the quantitative data, K-S test was used to analyze whether they were conformed to normality. Independent samples t-test was used for quantitative data that conformed to normal distribution, while chi-square test and fisher’s exact test were used for qualitative data and those that did not conform to normal distribution, and bilateral p<0.05 was considered to be statistically significant. The R packages used were: (1) “pROC” package for the ROC curves, (2) “rms” package for the column plots and calibration curves, (3) “glmnet” package for LASSO regression, (4) the “rmda” package for performing DCA, (5) the “shapviz” and “DALEX” packages for SHAP visualization and (6) the “psych” package for feature distribution of the model. All packages can be downloaded at https://cran.r-project.org/web/packages/. The Python packages used were (1) sklearn, which could be used for ML model construction and ROC curve plotting, and (2) PyRadiomics, which was used for the extraction of radiomics features.

## 3 Results

### 3.1 Patient distribution and clinical features selection

Based on a 0.7 ratio, 139 patients were randomly assigned to training and 59 to testing groups. **Table 1** gives baseline data for each set. The univariate analysis of the training set showed statistical significance for age, gender, SBP, PP, vulnerable plaque, degree of carotid stenosis, and TyG index. Clinical characteristics were then analyzed using multivariate LR with statistical significance for age, vulnerable plaque, carotid stenosis, and TyG score (**Table 2**).

**Table 1:** Baseline table.

Clinical variables	Training set (n = 139)			Testing set (n = 59)		
	No stroke (n = 73)	Stroke (n = 66)	*p1*	No stroke (n = 31)	Stroke (n = 28)	*p2*
Age, years	60.71 ± 9.96	66.41 ± 10.27	0.001*	59.93 ± 10.86	67.97 ± 8.75	0.003
Sex, male/female	33/40	43/23	0.018*	19/9	19/12	0.606
Current smoker, Y/N	17/56	16/50	0.896	6/22	8/23	0.699
Current drinker, Y/N	9/64	9/57	0.820	2/26	4/27	0.473
Living place, city/countryside	39/34	30/36	0.353	15/13	14/17	0.527
BMI	24.71 ± 3.47	24.37 ± 2.72	0.507	24.43 ± 2.54	24.68 ± 3.04	0.729
T2DM history †, years	10.0 (0.5-13.0)	7.5 (2.0-13.0)	0.965	10.0 (0.4-16.5)	9.0 (4.0-13.0)	0.876
Hypertension, Y/N	58/15	57/9	0.285	14/14	29/2	0.001
SBP (mmHg)	138.85 ± 21.41	146.27 ± 20.59	0.039	136.46 ± 20.24	151.00 ± 18.19	0.005
DBP (mmHg)	85.32 ± 15.37	83.11 ± 9.56	0.317	82.64 ± 10.85	83.87 ± 11.11	0.67
PP (mmHg)	53.53 ± 16.73	63.17 ± 18.97	0.002	53.82 ± 18.08	67.13 ± 13.98	0.002
Vulnerable carotid arteries plaques, Y/N	13/60	38/28	<0.0001*	5/23	17/14	0.003
Carotid Stenosis, Y/N	30/43	57/9	<0.0001*	12/16	25/6	0.002
Absent, n	43	9	–	16	6	–
Low, n	27	29	–	9	14	–
Moderate, n	3	20	–	2	5	–
Severe, n	0	8	–	1	6	–
LDL-C (mmol/L)	2.75 ± 1.11	2.59 ± 0.85	0.338	2.73 ± 0.92	2.67 ± 1.00	0.829
HDL-C (mmol/L)	1.09 ± 0.38	1.03 ± 0.37	0.351	1.06 ± 0.32	1.00 ± 0.35	0.516
FBS (mmol/L)	7.44 ± 2.67	7.44 ± 2.74	0.999	7.17 ± 2.86	7.03 ± 2.46	0.835
TC (mmol/L)	4.80 ± 1.53	4.42 ± 1.08	0.100	4.40 ± 1.09	4.73 ± 1.23	0.290
TG (mmol/L)	1.76 ± 2.22	1.76 ± 0.74	0.997	1.34 ± 0.52	2.18 ± 1.52	0.008
TyG index	8.89 ± 0.63	9.12 ± 0.52	0.010*	8.81 ± 0.65	9.18 ± 0.72	0.042

†medium (Q2-Q3); *p<0.05 in both univariate analysis and multivariate logistic regression.

**Table 2:** Multivariate Logistic regression of clinical features.

Variables	B	OR value	95%CI		*p*
			Lower limit	Upper limit	
Age	0.042	1.043	1.004	1.083	0.031*
Vulnerable carotid arteries plaques	0.834	2.301	1.000	5.301	0.049*
Carotid Stenosis	1.111	3.036	1.811	5.091	<0.0001*
SBP	0.007	1.007	0.977	1.039	0.638
PP	0.008	1.009	0.973	1.046	0.648
TyG index	0.843	2.324	1.256	4.300	0.007*
Sex	-0.434	0.648	0.303	1.385	0.262

*p < 0.05.

### 3.2 ML model selection and radiomics features selection and RS calculation

Labels with ICC<0.75 were excluded (**Supplementary Table ICC**), and 93 radiomics characteristics were chosen for T-test analysis. Features with p>0.05 were excluded. Eventually, 10 radiomics features were kept that were statistically significant between the study and control groups.

**Figure 3:**
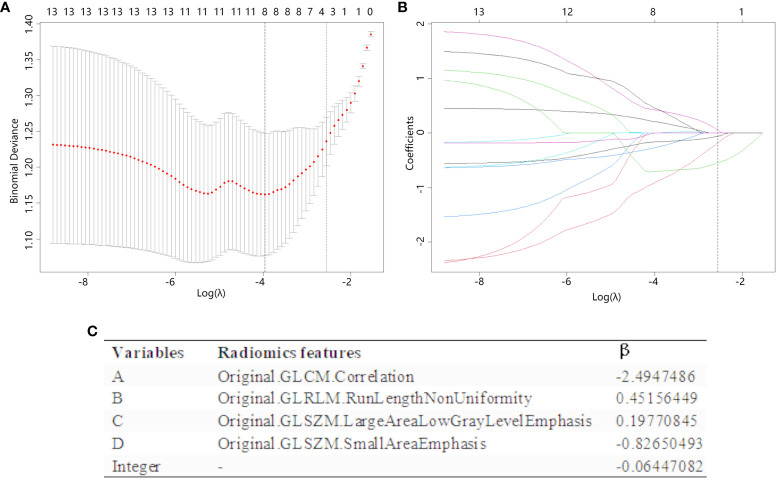
The radiomics features are chosen using LASSO regression. **(A)** The λ value in the LASSO regression is selected by 10-fold cross-validation, with the optimal value determined based on the 1-SE criterion. The figure displayed a vertical dashed line (right line) indicating the optimal value of λ, which was determined to be 0.0632. **(B)** The LASSO coefficient profiles of the 10 radiomics features that past the T-test show how the coefficients of the features changed in LASSO regressions as the value of λ was varied. At the optimal λ values, a total of four features with non-zero coefficients were selected. **(C)** Radiomics features selected by LASSO regression and their coefficients.

The radiomics features retained by the T-test was selected by LASSO regression (**Figure 3**), and a total of 4 features were selected when taking 1-standard error criterion (λ=0.0632), and **Figure 3C** demonstrates the variables and their corresponding coefficients in the LASSO regression. RS were constructed based on the four coefficients (β), which was calculated as RS= -0.06447082+(-2.4947486* A) + (0.45156449* B) + (0.19770845* C) + (-0.82650493* D).

Using these radiomics features, three ML models (SVC, RF, and LR) were created to find the best ML classifier. The three models’ ROC curves and the evaluation table were showed in **Supplementary Figure 1**, **Supplementary Table 2**, respectively. that LR model had the most stable performance in training and testing set and the biggest AUC in testing set, so the LR ML model would be used for modelling.

### 3.3 The construction and evaluation of the nomogram

RS calculated above was statistically significant between the study and control groups in the training and testing sets (**Supplementary Table 3**), and thus could continue to construct the radiomics +clinical combined LR model.

**Figure 4:**
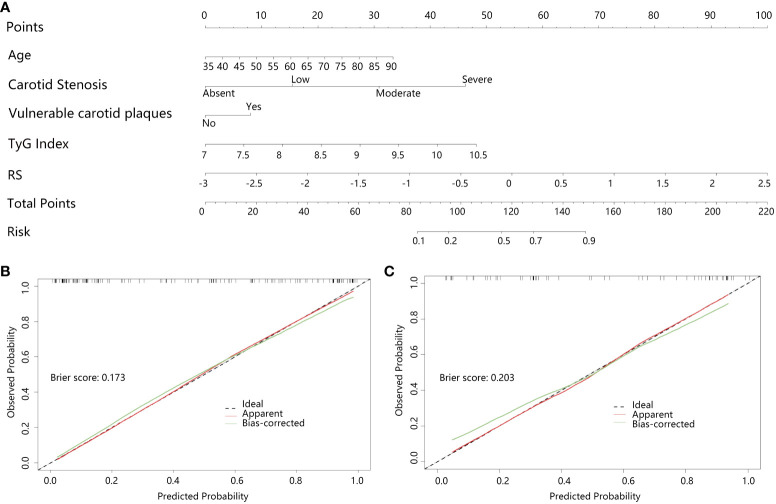
Clinical+radiomics nomogram and effectiveness test; **(A)** Clinical+radiomics nomogram, **(B, C)** are the calibration curves of the nomogram model in the training and testing set, respectively. The calibration curves indicate the goodness-of-fit of the nomogram. The diagonal line indicates the perfect match between the actual (Y-axis) and the predicted (X-axis) probabilities of the nomogram in the most ideal state. It showed that the Bias-corrected curves of the model were very close to the diagonal line in both the training and validation sets with the brier scores of 0.173 and 0.203, respectively, indicating that the model was highly accurate.

The radiomics nomogram (**Figure 4A**) combined the RS score of radiomics with the age, vulnerable carotid arteries plaques, carotid artery stenosis grade and TyG index, and the Nomo-score was calculated by the radiomics +clinical combined LR model as follow: Nomo-score = -11.87900300 + (1.24506418 * RS) + (0.03984024 * Age) + (1.03635580 * carotid stenosis) + (0.53637442 * vulnerable carotid arteries plaques) + (0.91575587 * TyG index), which was also statistically significant in both the training and testing sets (**Supplementary Table 3**). The calibration curves and the brier scores of the Nomogram model in the training set and the testing set were showed in **Figures 4B, C**. The feature distribution of the nomogram model was calculated and shown in **Supplementary Figure 2**.

### 3.4 Evaluation of the ML models

**Figure 5:**
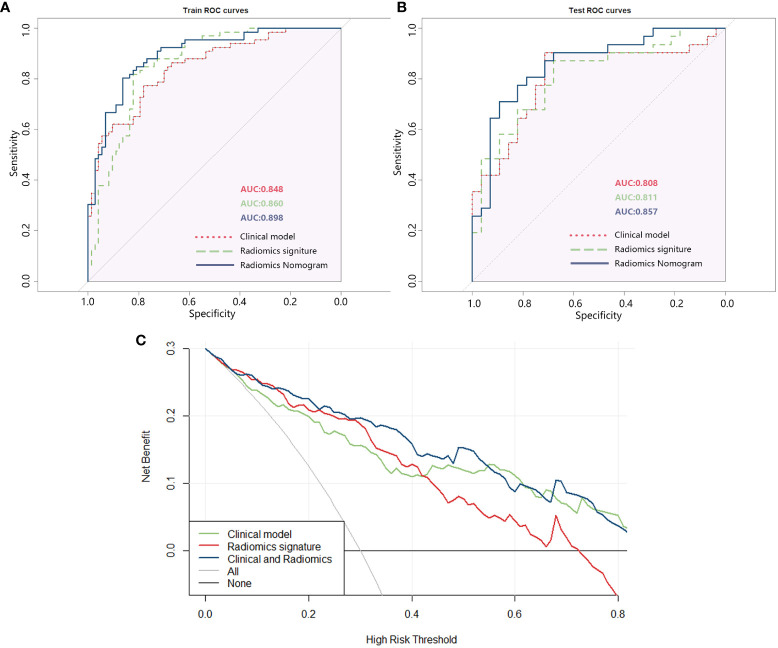
**(A)** ROC of the three models in the training set; **(B)** ROC of the three models in the testing set; **(C)** DCA curve of the three models.

The effectiveness of the three models (clinical model, radiomics model, and radiomics nomogram) was assessed individually and presented in **Supplementary Table 4** along with the ROC curve (**Figure 5**). To ensure each variable exhibits lower multicollinearity, the Variance Inflation Factors (VIF) were calculated between the variables in the radiomics nomogram, and the results were shown in **Supplementary Table 5**. The ROC analysis indicated that the radiomics nomogram (combined model) had the largest AUC among the three models in both the training and testing sets.

The DCA analysis of the three models (**Figure 5C**) revealed that the combined clinical + radiomics model (radiomics nomogram) had a higher overall net benefit compared to the other models in predicting the occurrence of IS in patients with T2DM, across most feasible threshold probability ranges.

**Figure 6:**
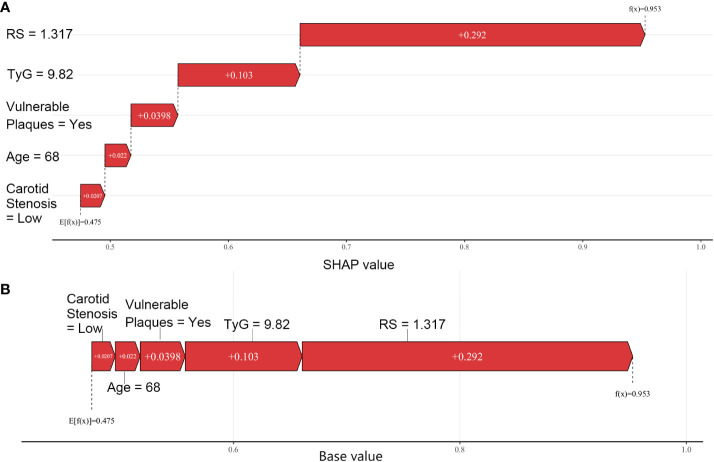
SHAP visualization for radiomics model; **(A)** Waterfall plot; **(B)** Force plot.

SHAP visualization of the radiomics nomogram model was shown in **Figure 6**. The waterfall diagram and the force plot (**Figures 6A, B**) display explanations for individual predictions of the radiomics nomogram model, in which RS plays an important role and TyG index comes next. All features display a positive contribution to the results.

## 4 Discussion

Assessing the risk of IS following the development of carotid plaque is crucial for the treatment plan for T2DM patients. This work aimed to build a radiomics nomogram based on CDU images using ML algorithm. The results of our study demonstrated that the nomogram shown a high level of diagnostic efficiency in predicting outcomes in both the training and testing sets (with an AUC of 0.898 in the training set and an AUC of 0.857 in the validation set). The brier scores of the calibration curves in the train set and test set was 0.173 and 0.203, respectively. This indicated that the radiomics nomogram we constructed had a high accuracy. Additionally, the DCA analysis confirmed that the radiomics nomogram created in our study could serve as a dependable clinical diagnostic tool for distinguishing the occurrence of IS in patients with T2DM.

Radiomics converts clinical images such as CT, MRI, and ultrasound images into radiomics features that can be combined with ML models to establish quantitative relationships between different types of data sources to identify and predict the risk of certain diseases (26). Researchers had developed a ML model to determine the presence or absence of symptoms based on carotid CTA (35, 36), and MRI radiomics can also be utilized to detect high-risk carotid artery plaque (37). However, there is currently a scarcity of predictive models using CDU imaging, which is a cost-effective and noninvasive diagnostic method for testing carotid plaques, to anticipate the occurrence of stroke symptoms. Previous studies have also confirmed the reliability of CDU in detecting vulnerable carotid plaques and the practicality of radiomics by comparing it with pathologic tissues (14, 15). In this study, our objective was to develop a more efficient and expedient prediction method. Through our analysis, we determined that LR exhibited superior performance out of another two ML models (including SVC and RF). Additionally, we utilized the radiomics of CDU images to construct a nomogram for predicting the risk of IS in T2DM patients. Our study specifically concentrated on patients with T2DM and introduced the TyG index to evaluate the impact of IR on the occurrence of IS. Our findings indicate that IR plays a significant role in predicting IS in individuals with T2DM.

Previous studies have confirmed that the severity of carotid stenosis and the composition of carotid plaque can predict the occurrence of IS. Our study supports these findings, as we found that both the degree of carotid stenosis (OR: 3.036, 95% CI: 1.811-5.091) and the presence of vulnerable plaque detected by CDU (OR: 2.301, 95% CI: 1.000-5.301) were associated with the occurrence of IS. Furthermore, the emerging TyG index has been noted to be associated with the presence of IS or coronary adverse cardiovascular events in previous studies (38–41). In addition, the association between elevated TyG index and vulnerable carotid plaque had also been noted (3, 23, 42). We found a correlation between the TyG index and the incidence of stroke events in T2DM patients (OR: 2.324, 95% CI: 1.256-4.300). Our study provides additional confirmation of the significance of the TyG index and IR in cardiovascular pathophysiology.

Interestingly, despite statistical significance in univariate analysis, SBP and PP were not statistically significant in multifactorial LR in our study, which differed from a previous clinical study conducted in China (43), and there is a consensus that hypertension is a risk factor for IS (5), despite our data performing in accordance with previous studies in univariate analysis. This nonsignificance in multifactorial LR could be attributed to the fact that timely management of T2DM patients following hypertension detection delayed disease development. Furthermore, lipid indices such as TG, LDL-C, and HDL-C were not significant in the univariate analysis of IS, which, while consistent with the findings of Kaze et al (44), was not consistent with the findings of Sun et al (45), who concluded in their study in a Chinese population that lowering LDL-C was likely to have a net benefit for the prevention of overall stroke and cardiovascular disease. We speculated that this was due to our patients’ routine use of cholesterol-lowering medicines, and because this was a cross-sectional retrospective study, prospective studies are still needed to investigate the association between lipid indices and IS.

Our study developed a nomogram based on CDU radiomics that can be used to predict IS risk in T2DM patients by identifying carotid plaque and corresponding clinical indicators (plaque nature, degree of carotid artery stenosis, TyG index size, and age), and validated the model’s reliability. Simultaneously, CDU was confirmed as a good method of cervical vascular plaque examination, and a nomogram prediction model was built in conjunction with the TyG index, another easily obtained index, to provide more personalized, convenient, and accurate stroke prevention and control measures for T2DM patients. By only need to obtain the TyG index and the CDU radiomics features, primary care physicians can predict a T2DM patient’s IS risk, and thus can personalize the medical support for them. One example of the application of the nomogram was shown in **Supplementary Figure 3**.

Despite the advantages of our study, there are also some limitations in our investigation. Firstly, this study was a retrospective analysis conducted on patients with T2DM. Due to ethical limitations, we were unable to request T2DM patients to discontinue their medication, and the method of obtaining image pictures posed challenges in conducting a prospective study to fully investigate the impact of carotid plaques on stroke development in the context of T2DM. Secondly, this study was conducted at a single center, and due to time constraints, we did not collect data from other institutions for a two-center validation. We will conduct additional external validation on external datasets in the future work.

## 5 Conclusion

In this study, based on CDU radiomics, a nomogram was constructed to identify IS risk in T2DM patients with a high diagnostic performance, which could be used in clinical diagnosis and provide accurate, convenient and personalized medical support for T2DM patients.

## Data availability statement

The original contributions presented in the study are included in the article/**Supplementary Material**. Further inquiries can be directed to the corresponding author.

## Ethics statement

The studies involving humans were approved by the medical ethics committee of the First Affiliated Hospital of Soochow University [Audit number: (2023) No.132]. The studies were conducted in accordance with the local legislation and institutional requirements. Written informed consent for participation was not required from the participants or the participants’ legal guardians/next of kin because this study was a retrospective analysis.

## Author contributions

YL: Investigation, Methodology, Software, Visualization, Writing – original draft, Writing – review & editing. YK: Formal Analysis, Software, Visualization, Writing – original draft, Writing – review & editing. YY: Conceptualization, Data curation, Project administration, Resources, Writing – review & editing. PH: Funding acquisition, Project administration, Resources, Supervision, Writing – review & editing.
