# Supplementary material for: Explore the value of carotid ultrasound radiomics nomogram in predicting ischemic stroke risk in patients with type 2 diabetes mellitus
Source: Front Endocrinol (Lausanne). 2024 Apr 19;15:1357580. doi: 10.3389/fendo.2024.1357580 (PMC11066235; doi:10.3389/fendo.2024.1357580)
Supplement: Supplementary file 1 [file DataSheet_1.docx]

**Supplementary Material**

Clinical characteristics were analyzed using multivariate logistic regression with statistical significance for age, vulnerable plaque, carotid stenosis, and TyG score (**STable 1**).

**STable 1 Multivariate Logistic regression of clinical features**

| Variables | B | OR value | 95%CI | | *p* |
| --- | --- | --- | --- | --- | --- |
|  |  |  | Lower limit | Upper limit |  |
| Age | 0.042 | 1.043 | 1.004 | 1.083 | 0.031* |
| Vulnerable carotid arteries plaques | 0.834 | 2.301 | 1.000 | 5.301 | 0.049* |
| Carotid Stenosis | 1.111 | 3.036 | 1.811 | 5.091 | <0.0001* |
| SBP | 0.007 | 1.007 | 0.977 | 1.039 | 0.638 |
| PP | 0.008 | 1.009 | 0.973 | 1.046 | 0.648 |
| TyG index | 0.843 | 2.324 | 1.256 | 4.300 | 0.007* |
| Sex | -0.434 | 0.648 | 0.303 | 1.385 | 0.262 |
| * *p* < 0.05 | | | | | |


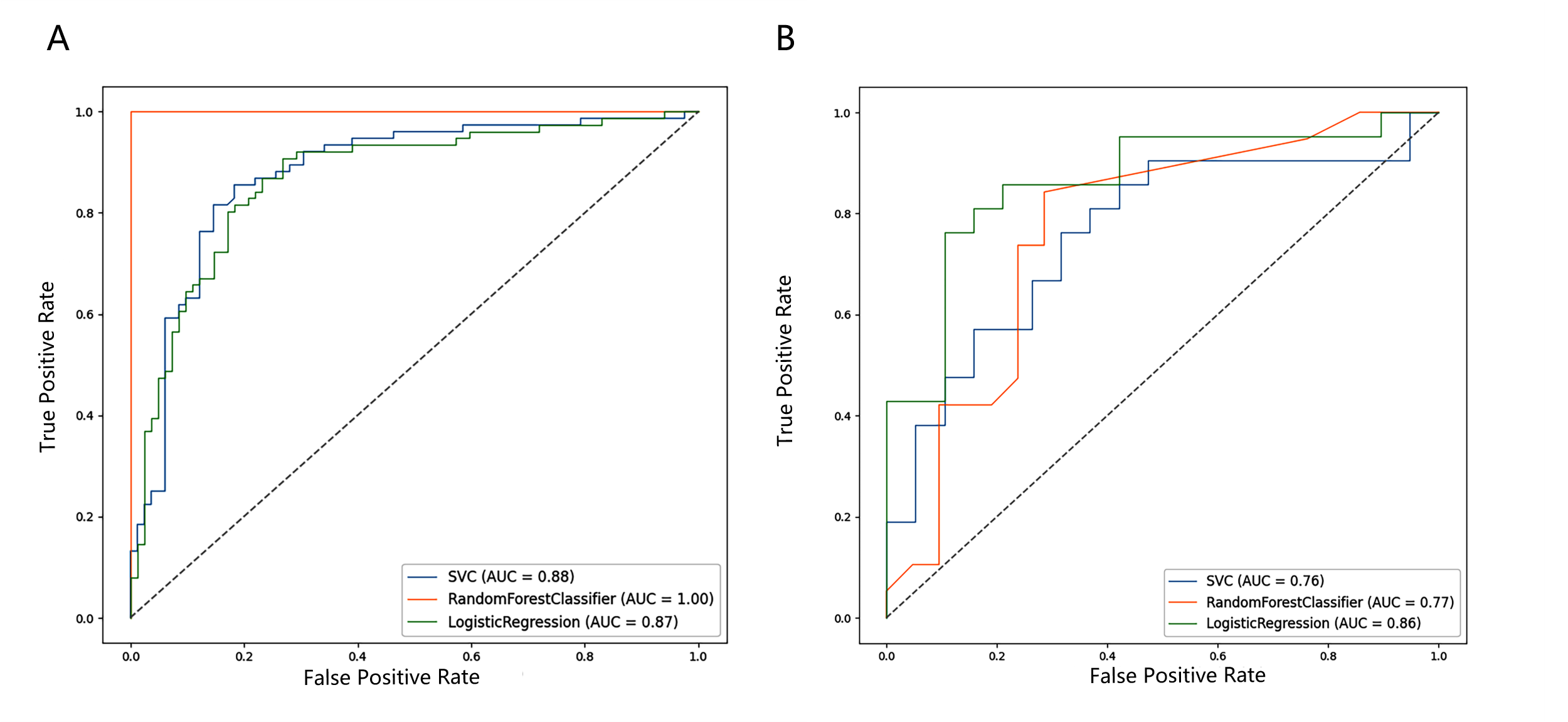
Selected radiomics features were utilized to construct and evaluate three ML models: Support Vector Classification (SVC), Random-Forest Classifier (RF), and Logistic Regression (LR). The effectiveness was evaluated and judged using receiver operating characteristic curves (ROC). The three models' ROC curves (**SFigure 2**) and the evaluation table (**Stable 2**) showed that LR model had the most stable performance in training and testing set and the biggest AUC in testing set.

**SFigure 1** ROC curves of preliminary ML models in the training and testing sets; A: ROC of the three ML models in the training set; B: ROC of the three ML models in the testing set

**STable 2 Evaluation of the three ML models**

| Model | Accuracy | Specificity | Precision | Recall (Sensitivity) | F1-score |
| --- | --- | --- | --- | --- | --- |
| SVC | 0.73 | 0.80 | 0.72 | 0.68 | 0.70 |
| RF | 0.65 | 0.58 | 0.64 | 0.76 | 0.70 |
| LR | 0.75 | 0.67 | 0.68 | 0.89 | 0.77 |

RS calculated above was statistically significant between the study and control groups in the training and testing sets (**STable 3**), and thus could continue to construct the radiomics +clinical combined LR model.

**STable 3 T-test results of RS and Nomo-score in training and testing set**

| Variables | Training set (n = 139) | | | Testing set (n = 59) | | |
| --- | --- | --- | --- | --- | --- | --- |
|  | No stroke (n = 73) | Stroke (n = 66) | *p1* | No stroke (n = 31) | Stroke (n = 28) | *p2* |
| RS | -1.066±0.234 | 0.666±0.307 | <0.0001 | -0.998±1.885 | 1.584±3.139 | <0.0001 |
| Nomo score | -2.084±2.639 | 1.715±2.939 | <0.0001 | -1.980±2.527 | 2.906±3.426 | <0.0001 |

The effectiveness of the three models (clinical model, radiomics model, and radiomics nomogram) was assessed individually and presented in **STable 4**

**STable 4: Relevant parameters for each model**

| Models | Accuracy | Specificity | Precision | Recall (Sensitivity) | F1-Score | AUC (95%CI) |
| --- | --- | --- | --- | --- | --- | --- |
| Training set |  |  |  |  |  |  |
| Radiomics model | 0.78 | 0.82 | 0.79 | 0.75 | 0.77 | 0.860 (0.798-0.922) |
| Clinical model | 0.76 | 0.79 | 0.76 | 0.71 | 0.73 | 0.848 (0.785-0.911) |
| Radiomics nomogram | 0.80 | 0.81 | 0.83 | 0.77 | 0.82 | 0.898 (0.847-0.949) |
| Testing set |  |  |  |  |  |  |
| Radiomics model | 0.80 | 0.68 | 0.77 | 0.82 | 0.77 | 0.811 (0.700-0.922) |
| Clinical model | 0.71 | 0.75 | 0.75 | 0.68 | 0.71 | 0.808 (0.691-0.924) |
| Radiomics nomogram | 0.83 | 0.82 | 0.80 | 0.85 | 0.80 | 0.857 (0.760-0.955) |

Variance Inflation Factors (VIF) were calculated between the variables in the radiomics nomogram, and the results were shown in **STable 5**. All variables included in the LR ML model showed low multicollinearity (VIF value < 10).

**STable 5: VIF of the variables in the radiomics nomogram model**

| Variables | Age | Carotid Stenosis | Vulnerable carotid arteries plaques | TyG index | RS |
| --- | --- | --- | --- | --- | --- |
| VIF | 1.059 | 1.141 | 1.164 | 1.070 | 1.081 |

**SFigure 2** showed the feature distribution in all patients and the correlation of the radiomics nomogram model. The upper half of the diagonals is the Pearson correlation matrix, the middle is the feature distribution histogram, and the lower half is the scatter matrix.


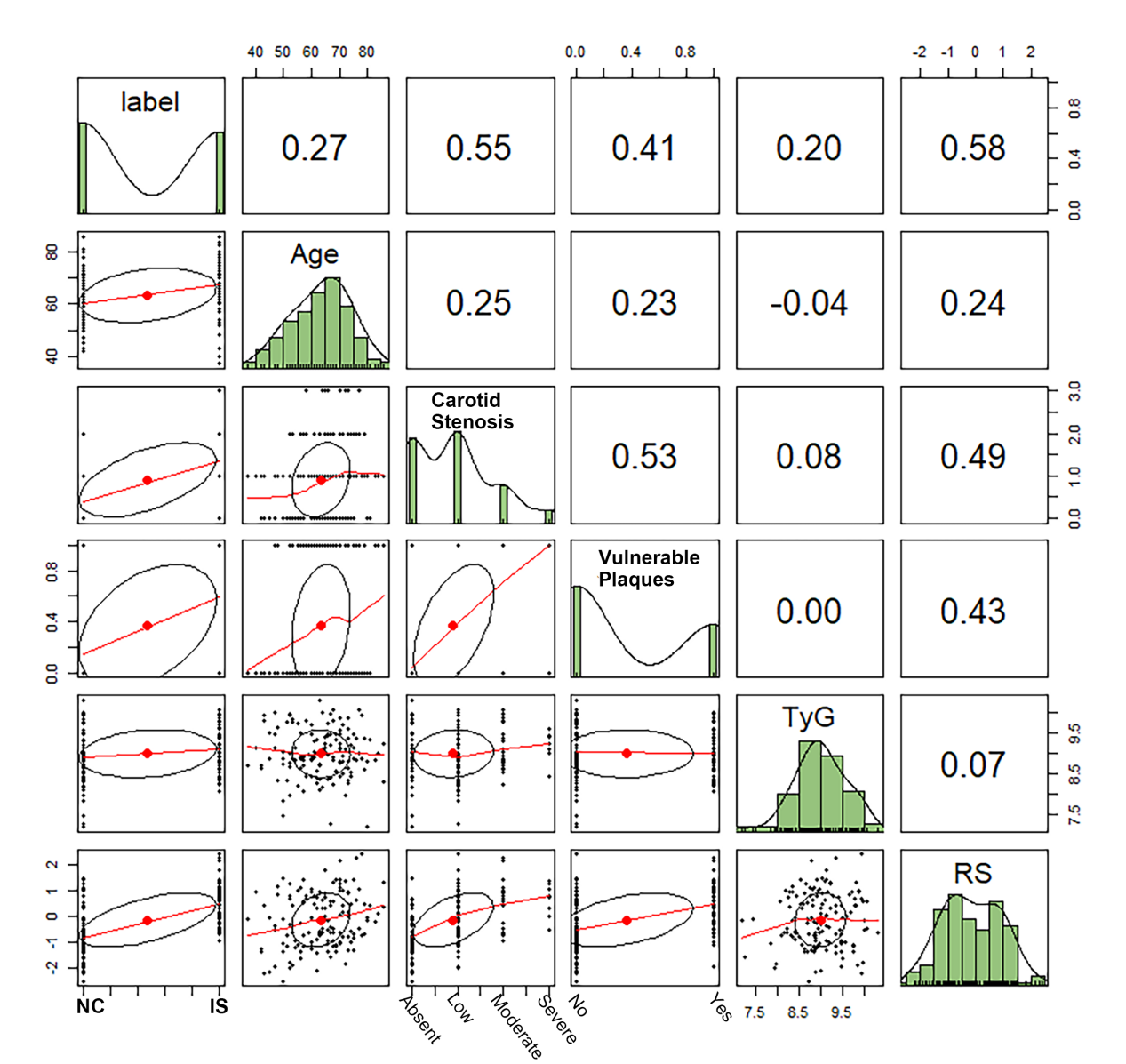


**SFigure 2** Feature distribution of the radiomics nomogram model. NC: Normal Control; IS: Ischemic Stroke; TyG: Triglyceride-glucose index


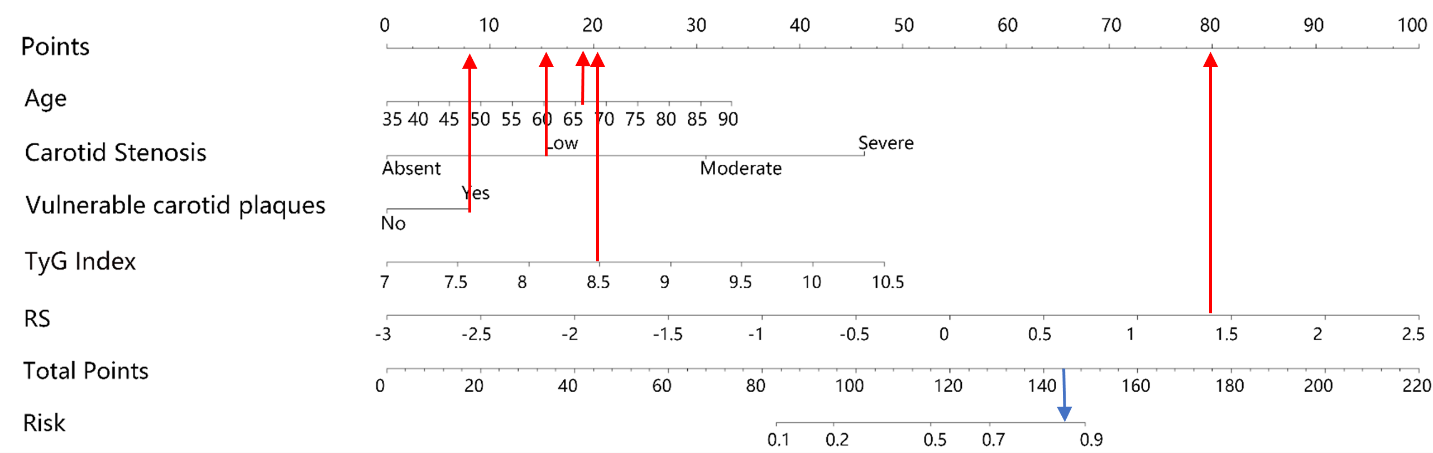
 An example of nomogram application: a 66-year-old male patient from the study's train set with low carotid stenosis and vulnerable plaque formation had an FBS of 6.33 and a TG of 0.95, resulting in a TyG of 8.48 calculated using the formula. His CDU RS was 1.44.The nomogram estimated a total of 142 points, and the probability of IS was calculated to be 88%. The genuine IS event occurred in this patient.

**SFigure 3** The application of the nomogram from this study
